# Mesenchymal Stem Cell Membrane-Coated TPCS_2a_-Loaded Nanoparticles for Breast Cancer Photodynamic Therapy

**DOI:** 10.3390/pharmaceutics15061654

**Published:** 2023-06-04

**Authors:** Greta Avancini, Luca Menilli, Adele Visentin, Celeste Milani, Francesca Mastrotto, Francesca Moret

**Affiliations:** 1 Department of Biology, University of Padova, Via Ugo Bassi 58/B, 35131 Padova, Italy; greta.avancini@phd.unipd.it (G.A.); luca.menilli@unipd.it (L.M.); celeste.milani@phd.unipd.it (C.M.); 2 Department of Pharmaceutical and Pharmacological Sciences, University of Padova, Via F. Marzolo 5, 35131 Padova, Italy; adelevisentin96@gmail.com

**Keywords:** biomimetic nanoparticles, photodynamic therapy, breast cancer

## Abstract

Despite substantial improvements in breast cancer (BC) treatment there is still an urgent need to find alternative treatment options to improve the outcomes for patients with advanced-stage disease. Photodynamic therapy (PDT) is gaining a lot of attention as a BC therapeutic option because of its selectivity and low off-target effects. However, the hydrophobicity of photosensitizers (PSs) impairs their solubility and limits the circulation in the bloodstream, thus representing a major challenge. The use of polymeric nanoparticles (NPs) to encapsulate the PS may represent a valuable strategy to overcome these issues. Herein, we developed a novel biomimetic PDT nanoplatform (NPs) based on a polymeric core of poly(lactic-co-glycolic)acid (PLGA) loaded with the PS meso-tetraphenylchlorin disulfonate (TPCS_2a_). TPCS_2a_@NPs of 98.89 ± 18.56 nm with an encapsulation efficiency percentage (EE%) of 81.9 ± 7.92% were obtained and coated with mesenchymal stem cells-derived plasma membranes (mMSCs) (mMSC-TPCS_2a_@NPs, size of 139.31 ± 12.94 nm). The mMSC coating armed NPs with biomimetic features to impart long circulation times and tumor-homing capabilities. In vitro, biomimetic mMSC-TPCS_2a_@NPs showed a decrease in macrophage uptake of 54% to 70%, depending on the conditions applied, as compared to uncoated TPCS_2a_@NPs. Both NP formulations efficiently accumulated in MCF7 and MDA-MB-231 BC cells, while the uptake was significantly lower in normal breast epithelial MCF10A cells with respect to tumor cells. Moreover, encapsulation of TPCS_2a_ in mMSC-TPCS_2a_@NPs effectively prevents its aggregation, ensuring efficient singlet oxygen (^1^O_2_) production after red light irradiation, which resulted in a considerable in vitro anticancer effect in both BC cell monolayers (IC_50_ < 0.15 µM) and three-dimensional spheroids.

## 1. Introduction

Breast cancer (BC) is one of the major health issues and the most common women’s cancer worldwide. Indeed, BC accounts for one in six female cancer-related deaths, mainly because of metastasis, recurrence, and drug resistance. Since the mid-2000s, its incidence has been steadily growing by about 0.5% per year, probably owing to the decline in fertility and continuous increase in body weight [1]. Therefore, the high and dangerous spread of the disease among the population, together with the poor survival rates observed, especially in the case of highly metastatic triple negative BC (TNBC) subtypes [2], demands that more effective treatment modalities are applied with or as alternatives to conventional BC therapeutic regimens (e.g., surgery, chemotherapy, radiation therapy).

Among clinically approved cancer treatments, photodynamic therapy (PDT) is a compelling cure for several solid tumors, able to efficiently reduce bulky cancer mass. PDT is based on three intrinsically non-toxic components, namely, a photosensitizer (PS), visible light, and molecular oxygen, which exert cytotoxicity only when combined. When the PS is activated with red light, it reacts with molecular oxygen at the site of accumulation, producing highly reactive oxygen species (ROS), mainly singlet oxygen (^1^O_2_), that causes local oxidative stress, cell death, and activation of immunological and inflammatory responses [3]. Even though it has not been as explored for other types of tumors such as lung, gastrointestinal, or bladder cancers [4,5], the potential use of a PDT regimen for BC cure is reported in many pre-clinical studies as well as in some clinical trials, primarily in patients with cutaneous or chest wall metastasis [6,7,8], rather than on primary tumors [9]. Although clinical research on PDT for BC is still at an early stage, its application seems promising since it has been observed that PDT localized treatment promotes immunity against recurrence and metastasis distant from the treated area, minimizing the patient’s collateral effects [10]. Indeed, one of the major advantages of PDT treatment relies on the concomitant activation of immunological processes, both locally at the irradiation site and distally, which significantly amplify ROS-induced cancer cell death. This activation is due to neutrophil infiltration in the tumor mass as well as improvement in tumor antigen presentation, T-cell activation, and suppression of Tregs, finally leading to tumor recurrence rejection [11]. Notwithstanding the abovementioned benefits, the major limitation to the widespread application of PDT is due to the hydrophobicity of the PSs that tend to aggregate in aqueous solution, thus making their administration rather difficult. Moreover, PSs are only partially selective toward neoplastic cells, a characteristic that significantly impairs the photodynamic activity [12]. Nanotechnological approaches applied to drug delivery have allowed the encapsulation of PSs in various nanoparticles (NPs), aiming at increasing the uptake by malignant cells and ameliorating the drug pharmacokinetic profile [13,14,15]. However, even if improved pharmacokinetic and reduced clearance have been observed for PS-loaded NPs in pre-clinical in vivo models [16], NP and therefore PS accumulation at the malignancy site is still quite poor, notwithstanding the contributions of the enhanced permeability and retention (EPR) effect [17] and of targeted nanosystems [18,19]. Thus, additional strategies must be identified to enhance the efficiency of PS-loaded NP accumulation in the tumor lesions, meanwhile considering all the other critical aspects of this type of cancer treatment, and to provide ad hoc tailored nano-drug formulations. Recently, biomimetic drug delivery platforms have gained a lot of interest, allowing researchers to build NPs endowed with functionality similar to that of biological components to promote an ameliorate soft matter interfacing with biomolecules, tissues, and living cells, coupled with the versatility of synthetic materials for payload retention and transport [20]. Therefore, plasma membranes extracted from different types of cells (e.g., red blood cells, platelets, macrophages, and cancer cells) have been used to camouflage the surface of drug-loaded NPs to prolong their circulation in the bloodstream, reduce the reticuloendothelial system’s clearance [21] and increase tumor targeting and accumulation [22]. Few authors have reported the successful application of the biomimicry technology for the selective delivery of PSs to tumors [23], including both homotypic [24,25] and heterotypic targeting [26,27].

Inspired by this tumor-targeting strategy, we generated a new biomimetic carrier for the administration of the PS meso-tetraphenylchlorin disulfonate (TPCS_2a_, also known as fimaporfin). TPCS_2a_ is an amphiphilic negatively charged PS approved by the FDA for photochemical internalization (PCI) technology [28]. TPCS_2a_-loaded NPs (TPCS_2a_@NPs) were generated using the highly biocompatible and biodegradable polymer poly(lactic-co-glycolic)acid (PLGA) and were synthesized with microfluidic technology, which allows a high batch-to-batch reproducibility, low reagent consumption, and narrows NP size distribution [29]. Through a simple process, TPCS_2a_@NPs were coated with mesenchymal stem cell-derived plasma membranes (mMSCs), resulting in a biomimetic nanocarrier (mMSC-TPCS_2a_@NP). The use of mMSCs to camouflage NPs was foreseen to avoid premature clearance by the mononuclear phagocytes system (MPS) and to promote innate migration toward the inflamed tumor site. Indeed, even though nanotechnology products have significantly improved pre-clinical outcomes in cancer therapy, clinical benefits and commercialization of nanopharmaceutics are still restricted to few examples (e.g., Abraxane, Doxil) owing mainly to sub-optimal tumor accumulation and rapid clearance. Stealth coating with PEG, the main strategy to prevent immune system recognition and clearance and to increase the plasma half-life of NPs, brings the drawback of acquired immunity that can cause a rapid clearance after multiple administrations [30]. As such, NP-based platforms employing biomimetic designs have attracted a lot of research attention [31]. MSCs were selected over other membrane typologies since it has been reported on their intrinsic homing ability toward injured and inflamed sites such as tumors, mainly thanks to the expression on their surface of multiple receptors for the recognition of specific molecules (e.g., C-X-C chemokine receptor type 4 (CXCR4), vascular endothelial growth factor receptor (VEGFR), and platelet-derived growth factor receptor (PDGFR)), released in the tumor microenvironment [32]). Considering that cancer is a disease characterized by chronic inflammation, MSCs have the potential to be a specialized therapeutic strategy to eradicate tumors, including BC.

## 2. Materials and Methods

### 2.1. Chemicals and Reagents

Poly(D,L-lactide-co-glycolide) (PLGA) (Resomer^®^ RG 503 H, Mw 24–38 kDa, 50:50), polyvinyl alcohol (PVA), the Spectra/Por^TM^ dialysis bag, and Amicon Ultra-4 Centrifugal Filters Unit with Ultracel were purchased from Merck (Darmstadt, Germany). Meso-tetraphenylchlorin disulfonate (TPCS_2a_, 2-MEA) was kindly provided by PCI Biotech AS (Oslo, Norway). Acetonitrile (MeCN), methanol (MeOH), and dimethylsulfoxide (DMSO) were purchased from Exacta Optech (Modena, Italy). Milli-Q water was produced by the Millipore Milli-Q^®^ purification system from Merck Millipore (Burlington, MA, USA). Cell culture medium (DMEM, DMEM/F12), grow factors, trypsin, antibiotics, horse serum, and fetal bovine serum (FBS) were purchased from Life Technologies (Milan, Italy). CellTiter96 Aqueous One Solution Cell proliferation Assay (MTS) and CellTiter-Glo 3D Cell Viability Assay were from Promega Co. (Madison, WI, USA). Cell culture plastic material was from Falcon (Corning, New York, NY, USA).

### 2.2. Cell Lines

ASC52telo (hTERT immortalized human adipose-derived mesenchymal stem cells), MCF7 (human breast adenocarcinoma), MDA-MB-231 (human triple negative breast cancer), and MCF10A (human non-tumorigenic breast epithelial cells) cell lines were purchased from American Type Tissue Culture Collection (ATCC, Rockville, MD, USA). All cells were maintained at 37 °C under a humidified atmosphere containing 5% CO_2_. ASC52telo cells were grown in Mesenchymal Stem Cell Basal Medium (ATCC) supplemented with 2% Fetal Bovine serum (FBS), 5 ng/mL rh FGF basic, 5 ng/mL rh FGF acidic, 5 ng/mL rh EGF, 2.4 mM L-Alanyl-L-Glutamine, 0.2 mg/mL G418, 10,000 U/mL penicillin G, 10 mg/mL streptomycin, and 25 ug/mL amphotericin B. MDA-MB-231 and MCF7 cells were grown using Dulbecco’s Modified Eagle Medium (DMEM) with Glutamax^TM^ supplemented with 10% FBS, 100 U/mL streptomycin and 100 µg/mL penicillin G (Life Technologies, Carlsbad, CA, USA). MCF10A were grown using DMEM/F12 supplemented with 5% horse serum (Life Technologies), 20 ng/mL EGF, 0.5 mg/mL hydrocortisone, 100 ng/mL cholera toxin, and 10 µg/mL insulin (Sigma Aldrich, Milano, Italy).

### 2.3. Synthesis of NPs

#### 2.3.1. Synthesis of TPCS_2a_-Loaded PLGA NPs (TPCS_2a_@NPs)

TPCS_2a_@NPs were manufactured via microfluidic mixing using the NanoAssemblr Benchtop instrument (Precision NanoSystems Inc., Vancouver, BC, Canada). Briefly, 50 µL of a 10 mg/mL TPCS_2a_ solution in DMSO was added to 950 µL of PLGA solution in MeCN to give a final concentration of 10 mg/mL PLGA and 0.5 mg/mL TPCS_2a_. An amount of 2% *w*/*v* PVA in Milli-Q water was used as the aqueous phase. The aqueous:organic flow rate ratio (FRR) was fixed at 1:1, and the total flow rate (TFR) was 12 mL/min. To remove the unloaded drug and the organic solvent, the aqueous NPs suspension was transferred to an Amicon Ultra-4 Centrifugal Filters Unit with Ultracel^®^ regenerated cellulose membrane with a 30 kDa molecular weight cut-off (MWCO). NPs were washed four times with Milli-Q water at 192× *g* at room temperature for 50 min. Afterwards, NPs were freeze-dried and the amount of TPCS_2a_ loaded in the NPs was quantified by an Evolution TM UV- Vis spectrophotometer (Thermo Fisher, Waltham, MA, USA) at 416 nm (ε_416_ = 201,000 L/(mol/cm)). One milligram of freeze-dried purified NPs was dissolved in DMSO (final concentration 10 mg/mL) and diluted to a suitable concentration in CH_3_OH. The encapsulation efficiency (EE) and the loading capacity (LC) of NPs were calculated according to Equations (1) and (2):

(1) $$\mathrm{EE}\left(\%\right)=\frac{\mathrm{Loaded}\;\mathrm{TPCS}2a\;\left(\mathrm{mg}\right)}{\mathrm{AddedTPCS}2a\;\left(\mathrm{mg}\right)}*100$$

(2) $$\mathrm{LC}\;\left(\%\right)=\frac{\mathrm{Loaded}\;\mathrm{TPCS}2a\;\left(\mathrm{mg}\right)}{\mathrm{TPCS}2a+\mathrm{PLGA}\;\left(\mathrm{mg}\right)}*100$$

#### 2.3.2. Isolation of Mesenchymal Stem Cell-Derived Plasma Membranes (mMSC)

Mesenchymal stem cell-derived plasma membranes (mMSCs) were isolated from ASC52telo cells following the method reported by Gao et al. [33]. Briefly, when the cells reached confluence, they were detached by trypsin-EDTA treatment and incubated with 40 µL of 3,3′-Dioctadecyloxacarbocyanine perchlorate (DiO) in DMEM for 20 min at 37 °C and 5% CO_2_ atmosphere. Afterwards, cells were suspended in a hypotonic lysing buffer (1 mM NaHCO_3_, 0.2 mM EDTA, and 1 mM protease inhibitor at 4 °C) and were enucleated in a hand-held Dounce homogenizer. Then, the supernatants were collected by centrifugation (3200× *g* for 5 min at 4 °C), and centrifuged again at 15,000× *g* for 30 min at 4 °C. The prepared isolated membranes were dispersed in PBS (pH 7.4) and stored at −80 °C for subsequent experiments. The protein content in the purified mMSCs was determined by BCA protein assay [34].

#### 2.3.3. Preparation of Biomimetic TPCS_2a_ NPs (mMSC-TPCS_2a_@NPs)

mMSCs isolated as previously described were mixed with TPCS_2a_@NPs (final PLGA concentration 2 mg/mL) in DNase and RNase free sterile water (final mMSCs and PLGA concentrations of 0.05 and 2 mg/mL, respectively). The mixture was ultrasonicated with an Omni Sonic Ruptor 250 (Omni International Inc., Kennesaw, GA, USA) for 5 min at 50 W with pulse to produce the biomimetic nanosystem (mMSC-TPCS_2a_@NPs). Finally, the aqueous suspension was centrifuged at 15,000× *g* for 30 min to collect mMSC-TPCS_2a_@NPs, which were resuspended in 200 µL of DNase and RNase free sterile water for the following experiments. As control, mMSCs were subjected to the same procedure in the absence of TPCS_2a_@NPs, resulting in mMSC vesicles.

### 2.4. Characterization of TPCS_2a_@NPs and mMSC-TPCS_2a_@NPs

#### 2.4.1. Size Distribution and Zeta Potential Analyses

The mean hydrodynamic diameter and the polydispersity index (PDI) of TPCS_2a_@NPs and mMSC-TPCS_2a_@NPs were measured at 25 °C by Dynamic Light Scattering (DLS) using the ZetaSizer Ultra (Malvern Instruments Ltd., Malvern, UK) equipped with a laser at a multi-angle. The zeta potential (ZP) measurements were performed by laser Doppler electrophoresis at an NP concentration of 1 mg/mL in 15 mM 4-(2-Hydroxyethyl)piperazine-1-ethanesulfonic acid (HEPES) buffer, pH 7.4. Data were analyzed using ZS XPLORER 2.0.1.1 software.

#### 2.4.2. Freeze-Drying Studies

TPCS_2a_@NPs were freeze-dried in the presence of different cryoprotectants such as dextrose, glucose, sucrose, and trehalose, using the freeze-dryer Hetossic HETO Lab Equipment (Birkerod, Denmark). Increasing volumes of 100 mg/mL sugar solutions in Milli-Q water were added to 200 µL aliquots of a 15 mg/mL TPCS_2a_@NPs aqueous suspension to achieve the final sugar concentrations of 1%, 3%, 5%, and 10% (*w*/*v*). Freeze-dried samples were then resuspended in Milli-Q water at a PLGA concentration of 2 mg/mL and analyzed by DLS. For each sample, the intensity-weighed mean value and PDI were recorded as the average of three measurements. Resuspended TPCS_2a_@NPs were purified to remove TPCS_2a_ possibly leaked during the process and the drug content was re-evaluated as described in Section 2.3.1, Synthesis of TPCS_2a_-loaded PLGA NPs (TPCS_2a_@NPs).

#### 2.4.3. Release Kinetic of TPCS_2a_ from TPCS_2a_@NPs

Free TPCS_2a_ or TPCS_2a_@NP suspension (50 µM drug equivalent concentration) in 10 mM phosphate buffer (PB), pH 7.4 was placed into a 10 kDa MWCO dialysis bag and dialyzed against 1 L of PB added of 0.05% (*w*/*v*) Tween 80 at 37 °C. At scheduled times 50 µL of samples were withdrawn, and drug content was detected by UV-Vis spectrophotometer analysis at 421 nm, after appropriate dilution in DMSO, according to a TPCS_2a_ calibration curve prepared in DMSO in the 0.1–5 µM concentration range (y = 0.2057x + 0.00468, R^2^ = 0.9877).

The drug release kinetic was fitted with the Weibull and the Korsmeyer–Peppas models characterized by Equations (3) and (4), respectively:

(3) $$M=M_{0}\;\left[1-e^{\frac{{\left(t-T\right)}^{b}}{a}}\right]$$

where M is the amount of drug dissolved, M_0_ is the total amount of drug released, T is the lag time, a is a scale parameter that describes the time dependence, and b is the shape of the dissolution curve,

(4) $$\frac{M_{t}}{M_{\infty }}={\mathrm{Kt}}^{n}$$

where M_t_/M_∞_ is the drug released at time t, k is the release rate constant, and n is the release exponent.

#### 2.4.4. SDS-PAGE

The protein content of whole ASC52telo cell extracts, purified mMSCs, and mMSC-TPCS_2a_@NPs was analyzed by sodium dodecyl sulfate-polyacrylamide gel electrophoresis (SDS-PAGE). All samples were lysed using RIPA buffer, centrifuged at 15,000× *g* for 15 min, heated at 95 °C for 5 min, and then loaded into a Bolt 4–12% Bis-Tris gel (Life Technologies, Milan, Italy). Samples were electrophoresed at 130 V for 1 h, using the electrophoresis apparatus Invitrogen Mini Gel Tank (Life Technologies), and then the proteins were stained with silver staining and visualized with Gel Doc XR+ System (Bio-Rad, Hercules, CA, USA) with ImageLab Software.

#### 2.4.5. Transmission Electron Microscopy (TEM)

TEM images were obtained with a Tecnai G2 microscope (FEI, Hillsboro, OR, USA). TPCS_2a_@NP, mMSC, and mMSC-TPCS_2a_@NP aqueous suspensions (2 mg/mL PLGA concentration) were placed on a carbon-coated copper grid and the water was allowed to dry at room temperature. NPs were negative stained using a 1% *w*/*v* uranyl acetate solution in distilled water. The average diameter of particles was calculated by measuring 50 individual particles with ImageJ Software v.1.51.

#### 2.4.6. Stability Studies

Stability studies of TPCS_2a_@NPs and mMSC-TPCS_2a_@NPs were carried out by diluting NPs to a final concentration of 2 mg/mL PLGA in Milli-Q water or DMEM, in the presence or absence of 5% of FBS. The final suspension was aliquoted and maintained at 4 °C or 37 °C up to 72 h for the aliquots in DMEM with serum and up to 7 days for those without serum. The mean hydrodynamic diameter and PDI of particles were analyzed at scheduled intervals by DLS.

#### 2.4.7. Spectroscopic Studies

To characterize the spectroscopic properties of TPCS_2a_ in different media as a free drug or loaded in TPCS_2a_@NPs or mMSC-TPCS_2a_@NPs, UV-Vis and fluorometric analyses were carried out using an Eppendorf BioSpectrometer^®^ basic (Eppendorf, Hamburg, Germany) and a Varian Cary Eclipse Fluorometer (Agilent Technologies Inc., Santa Clara, CA, USA), respectively. TPCS_2a_ prepared in a DMSO solution, TPCS_2a_@NPs or mMSC- TPCS_2a_@NPs prepared as described in Section 2.3, Synthesis of NPs, were diluted in Milli-Q water or DMEM added of 10% of FBS to a final TPCS_2a_ concentration of 2.5 µM. Samples were maintained at 4 °C or 37 °C and absorbance and fluorescence spectra were registered at scheduled times up to 24 h. All UV-Vis spectra were recorded in the 300–750 nm range, and for the fluorescence spectra the excitation wavelength was set at 550 nm and emission recorded in the 600–700 nm range.

#### 2.4.8. Singlet Oxygen (^1^O_2_) Generation

^1^O_2_ generated by free TPCS_2a_, TPCS_2a_@NPs, or mMSC-TPCS_2a_@NPs was determined by singlet oxygen sensor green (SOSG) reagent. Free TPCS_2a_ in DMSO, TPCS_2a_@NPs, or mMSC-TPCS_2a_@NPs was dissolved in H_2_O and mixed with SOSG to obtain a TPCS_2a_ equimolar concentration of 2 µM and SOGS concentration of 10 µM. These solutions were irradiated with red light (600–800 nm) emitted from a Waldmann PDT 1200 lamp up to 10 min. The power density was 6.5 mW/cm^2^ as measured with the radiometer PDT1200 (Waldmann, Germany). Fluorescence intensities were recorded using a λ_ex_ of 488 and a λ_em_ of 527 nm. The percentage increase in fluorescence at 527 nm over time was calculated according to the following Equation (5):

(5) $$\mathrm{Increase}\;\left(\%\right)=\left[I\;\left(t\right)-\frac{I\;\left(t_{0}\right)}{I\;\left(t_{10}\right)}\right]\times 100$$

where I (t) is the intensity measured at each time point, I (t_0_) is the intensity measured at time 0 and I (t_10_) is the intensity measured at t_end_ (10 min).

### 2.5. NP Capture by Human Macrophages

#### 2.5.1. Isolation and Differentiation of Monocytes from Buffy Coat

Human macrophages were obtained from human monocytes purified from buffy coats of healthy donors (provided by the Transfusion Center of the Hospital of Padova) by Ficoll and Percoll gradients following a protocol adapted in our laboratory [35]. Monocytes were differentiated into macrophages by incubation in RPMI added with 20% of FBS and 100 ng/mL of human macrophage colony-stimulating factor (M-CSF) for 7 days.

#### 2.5.2. NP Internalization by Human Macrophages

Seven days old macrophages were incubated for 2 h in RPMI containing 0.5 µM TPCS_2a_ NPs at 37 °C. In some cases, TPCS_2a_@NPs or mMSC-TPCS_2a_@NPs were pre-incubated in complete human serum (HS) for 30 min or 2 h at 37 °C before the addition to the cells. Afterwards, macrophages were washed twice with PBS w/o Ca^2+^ and Mg^2+^ and detached with 500 µL of 5 mM EDTA in PBS. Macrophages were collected by gently pipetting, centrifuged at 200× *g*, and resuspended in Versene. Samples were analyzed on a BD Fortessa^TM^ cytofluorimeter using the red laser (640 nm) to excite TPCS_2a_ and detecting the fluorescence signal on the APC channel (λ > 660 nm). A minimum of 10^4^ events were acquired per sample and data were analyzed using FACSDiva Software.

### 2.6. In Vitro Studies on BC Cell Monolayers

#### 2.6.1. Uptake Studies

MDA-MB-231, MCF7, and MCF10A cells were seeded at the density of 5 × 10^4^ cells/well in 24-well plates, grown overnight, and incubated with 0.125 or 0.25 µM of free TPCS_2a_ or equimolar concentrations of TPCS_2a_@NPs and mMSC-TPCS_2a_@NPs in complete medium for 24 h. At the end of the incubation time, cells were washed twice with 500 µL of Versene, detached from the plate with 250 µL of a 0.25% trypsin solution in PBS and added with 200 µL of FBS. Cells were collected by gently pipetting, centrifuged at 200× *g*, and resuspended in Versene. Samples were analyzed on a BDFortessa cytometer using the same set-up described above.

#### 2.6.2. Confocal Microscopy Uptake and Localization Studies

The intracellular uptake of biomimetic NPs in which mMSCs were previously stained with DiO was also investigated by confocal microscopy to assess if the membrane coating remains associated to the NP/PS (distinguishable by the red fluorescence of TPCS_2a_) even after endocytosis. Therefore, MCF7 cells were seeded onto 35 mm imaging dishes (Eppendorf) at the density of 8 × 10^4^ cells/dish in DMEM added of 10% FBS (DMEM complete medium) and incubated for 24 h at 37 °C with 1 µM of mMSC-TPCS_2a_@NPs or TPCS_2a_@NPs as control. Cells were then washed with PBS, fixed with 4% paraformaldehyde (PFA), and imaged on a Leica SP5 inverted confocal laser-scanning microscope equipped with a 488 nm Ar laser, 543/633 HeNe laser, and 40×/0.7 NA objective. The images were elaborated using LAS X software (Leica Microsystems GmbH, Wetzlar, Germany). For intracellular localization studies, MDA-MB-231 cells were seeded as described above and, at the end of the growing period, the medium was replaced with fresh medium containing 1 µM free TPCS_2a_ or equimolar concentrations of TPCS_2a_@NPs and mMSC-TPCS_2a_@NPs, and cells were incubated for 24 h at 37 °C. Fifteen minutes before the end of incubation, a 50 nM LysoTracker Green DND-26 solution and 10 µM of Hoechst 33342 solution were added to the cells. Cells were then washed twice with PBS with Ca^2+^ and Mg^2+^. The images were obtained with the same instrument described above.

#### 2.6.3. Dark and Phototoxicity Studies

The in vitro dark and phototoxicity of free TPCS_2a_, TPCS_2a_@NPs, and mMSC-TPCS_2a_@NPs were measured by the MTS assay (CellTiter 96 AQueous One Solution Cell Proliferation Assay). For dark cytotoxicity, MDA-MB-231 and MCF-7 cells were seeded at the density of 1 × 10^4^ cells/well in a 96-well plate in DMEM complete medium. After 24 h, the medium was replaced with fresh medium containing increasing concentrations of free TPCS_2a_ or equimolar concentrations of TPCS_2a_@NPs and mMSC-TPCS_2a_@NPs, and cells were incubated for 24 h at 37 °C. Afterwards, the medium was replaced with fresh complete medium and cell viability was measured after a further 24 h of incubation. For phototoxicity experiments, after 24 h of incubation with the different treatments, cells were washed twice with 150 µL of PBS with Ca^2+^ and Mg^2+^ and irradiated with red light (600–800 nm) emitted from a Waldmann PDT1200 lamp with a total fluence of 10 J/cm^2^. The power density was 40 mW/cm^2^ as measured with the radiometer PDT1200 (Waldmann, Germany). After irradiation and replacement of PBS with fresh medium, cells were incubated for 24 h in the dark before performing cell viability measurements. For the MTS assay, the medium was discarded and 100 µL of serum-free DMEM medium and 20 µL of CellTiter 96^®^ were added to each well and the plates were incubated for 45 min at 37 °C in the dark. Then, the absorbance of each well was measured at 492 nm by a Multiskan Go plate reader (Thermo Fisher, Waltham, MA, USA) and cell viability was expressed as percentage of the absorbance with respect to control cells (100% viability). The drug concentration that inhibits cell survival by 50% (IC_50_ value) was calculated by the software GraphPad Prism 7 v.7.04.

### 2.7. In Vitro Studies on Spheroids

#### 2.7.1. Generation of Multicellular Tumor Spheroids

Spheroids of MDA-MB-231 cells were generated using the liquid overlay method, as previously reported by our group [36]. Cells were harvested from monolayer cultures by trypsinization, resuspended in DMEM complete medium added with 7.5 µg/mL of collagen, and 3.5 × 10^3^ cells/well were seeded in a 96-well Ultra-Low Attachment (ULA) plate (Nuclon Sphera U-shaped bottom microplates, Thermo Fisher Scientific). Immediately after seeding, the plates were centrifuged at 100× *g* for 3 min to promote cell aggregation and then placed in the incubator at 37 °C and 5% CO_2_.

#### 2.7.2. Dark and Phototoxicity Studies

The in vitro cyto- and phototoxicity on spheroids of free TPCS_2a_, TPCS_2a_-@NPs, and mMSC-TPCS_2a_@NPs were measured using CellTiter-Glo^®^ 3D Cell Viability Assay or LIVE/DEAD^TM^ Viability/Cytotoxicity Kit. Four days old spheroids were incubated with 100 µL of DMEM complete medium at increasing concentrations (0.05–0.5 µM) of free TPCS_2a_ or equimolar concentrations of TPCS_2a_@NPs and mMSC-TPCS_2a_@NPs for 24 h at 37 °C.

For phototoxicity experiments, after 24 h of incubation with PS formulations, spheroids were washed twice with 100 µL of DMEM w/o phenol red and irradiated with red light (600–800 nm) emitted from a Waldmann PDT1200 lamp with a total fluence of 10 J/cm^2^ (power density was 40 mW/cm^2^). After irradiation, spheroids were incubated for 24 h in the dark before the 3DGlo assay. A total of 100 µL of CellTiter-Glo 3D Reagent was added to 100 µL of cell medium, the well content was mixed, and the plate was incubated for 30 min at room temperature in the dark. Then, the luminescence signal of each sample was measured by Victor X3 (PerkinElmer, Waltham, MA, USA) and the viability of spheroids was expressed as a percentage of the luminescence with respect to control cells (100% viability). The drug concentration that inhibits cell survival by 50% (IC_50_ value) was calculated by the software GraphPad Prism 7 v.7.04.

For LIVE/DEAD experiments, after 24 h of incubation with PS formulations, the medium was replaced with fresh complete medium and spheroid viability was measured after a further 24 h of incubation. In all samples, 100 µL of 2 µM calcein AM and 4 µM ethidium homodimer solutions in PBS were added to 100 µL of cell medium and spheroids were incubated for 30 min at 37 °C in the dark. Afterwards, the dyes were removed, and the spheroids were washed with 150 µL of PBS. The images were obtained on a Leica DMI4000B fluorescence inverted microscope equipped with a 5×/0.12 NA objective (Leica Microsystems GmbH, Wetzlar, Germany).

#### 2.7.3. Uptake Studies

Four days old spheroids were incubated for 24 h with 100 µL of DMEM complete medium containing 1 µM free TPCS_2a_ or equimolar concentrations of TPCS_2a_@NPs and mMSC-TPCS_2a_@NPs. Then, the spheroids were washed twice with 100 µL of DMEM w/o phenol red and images were acquired on a Leica SP5 inverted confocal laser-scanning microscope equipped with a 488 nm Ar laser, 543/633 HeNe laser, and 40×/0.7 NA objective. Images were acquired from the top to the equatorial plane of the spheroid in about 50 different focal planes and elaborated with the LAS X software (Leica) to create the images with the superimposition of all the acquired focal planes.

### 2.8. Statistical Analysis

All the results were expressed as mean ± standard deviation (SD). Calculations and comparisons between groups were performed with the software GraphPad Prism 7 v.7.04 using one-way or two-way ANOVA. *p*-values ≤ 0.05 were considered significant.

## 3. Results and Discussion

### 3.1. Preparation and Characterization of mMSC-TPCS_2a_@NPs

The main challenges associated with PDT are the lack of PS solubility in aqueous media and their scarce selectivity for cancer cells. Thus, we herein designed unprecedented biomimetic poly(lactic-co-glycolic) (PLGA) NPs loaded with the PS TPCS_2a_ for breast cancer photodynamic therapy (BC-PDT).

**Figure 1:**
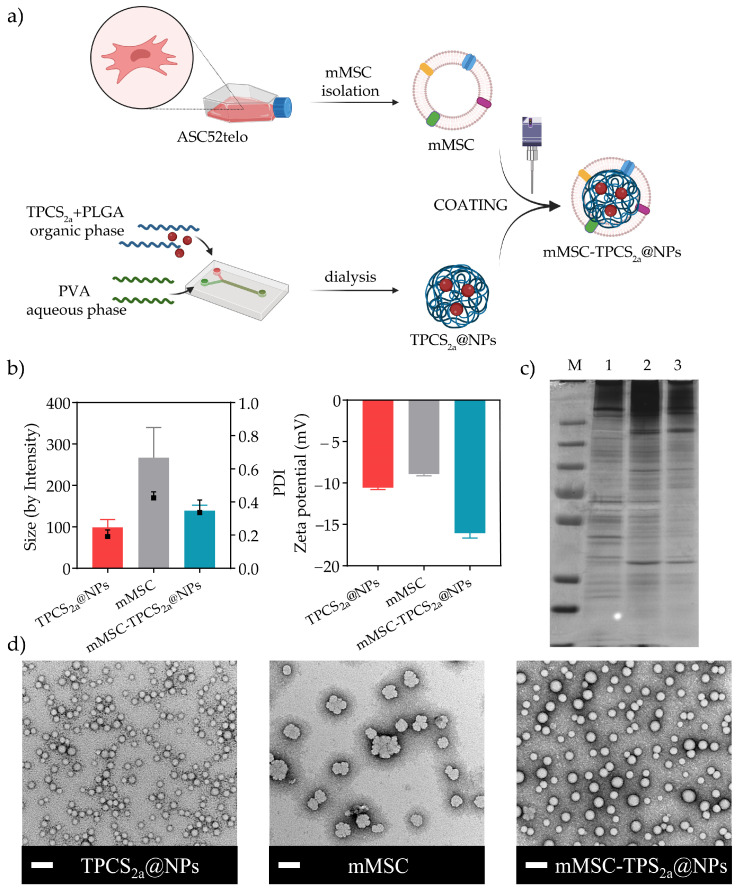
Formulation scheme and characterization of TPCS_2a_@NPs and mMSC-TPCS_2a_@NPs. (**a**) Scheme illustrating the formulation procedure of mesenchymal stem cell-coated TPCS_2a_@NPs (mMSC-TPCS_2a_@NPs). (**b**) Hydrodynamic diameter, polydispersity index (PDI), and zeta potential (ZP) of TPCS_2a_@NPs, mMSC vesicles, and mMSC-TPCS_2a_@NPs. (**c**) Representative silver-stained SDS-PAGE protein pattern of: line 1 = ASC52telo cells lysate, line 2 = isolated mMSC, line 3 = mMSC-TPCS_2a_@NPs, M = protein marker. (**d**) Representative transmission electron microscopy (TEM) images of TPCS_2a_@NPs, mMSC vesicles, and mMSC-TPCS_2a_@NPs; scale bars: 100 nm.

As illustrated in Figure 1a, the formulation of mMSC-TPCS_2a_@NPs involved three steps: (i) assembling of TPCS_2a_-loaded PLGA NPs (TPCS_2a_@NPs); (ii) isolation of mMSCs from ASC52telo mesenchymal stem cells; (iii) coating of TPCS_2a_-loaded PLGA NPs with mMSCs.

TPCS_2a_@NPs were produced via microfluidic technique leading to NPs of a well-defined diameter and size distribution of 98.89 ± 18.56 nm and 0.191 ± 0.04, respectively, as measured by DLS (Figure 1b). Importantly, after purification the encapsulation efficiency % (EE) resulted as high as 81.90 ± 7.92%. This was particularly relevant considering the very low solubility of the drug in the solvent used for the NP formulation, and thus the limited loading capacity % (LC) expected, which was found to be 1.50 ± 0.26%. Despite that, the amount of drug entrapped into the NPs was considered adequate for a clinical translation owing to the elevated activity of TPCS_2a_, as previously demonstrated by our group [37,38].

Next, we coated TPCS_2a_@NPs with mMSC isolated as previously described by Gao et al. [31]. Among the different options currently employed to coat NPs with cell membrane material, including extrusion and microfluidic electroporation, we selected sonication. Sonication is a convenient and time-saving method that allows for the reduction of the waste resulting from the build-up material on the porous membrane when extrusion is employed [39]. The sonication of the suspension allowed physically disassembling the cell membranes followed by their reassembling around the polymeric core, generating mMSC-coated TPCS_2a_@NPs (mMSC-TPCS_2a_@NPs). As control, mMSC vesicles were produced by applying the same methodology to the isolated membrane alone.

The generated TPCS_2a_@NPs, mMSC vesicles, and mMSC-TPCS_2a_@NPs were characterized for their hydrodynamic diameter, polydispersity index (PDI), and zeta potential (ZP) (Figure 1b). The hydrodynamic diameter of TPCS_2a_-loaded NPs increased from 98.89 to 139.31 ± 12.94 nm after membrane coating, suggesting the efficiency of the coating procedure. The high reproducibility attained by the microfluidic approach was corroborated by the consistency of the size values obtained among different preparations, as shown by the very low size standard deviation. The disappearance after sonication of the population detected by DLS at 98.89 ± 18.56 nm and at 266.73 ± 72.75 nm attributed to TPCS_2a_@NPs and mMSC vesicles, respectively, and the appearance of a new peak at 139.31 ± 12.94 nm with a PDI of 0.336 ± 0.076 further confirmed the procedure was successful. To visualize the mMSC coating and compare TPCS_2a_@NPs, mMSC vesicles, and mMSC-TPCS_2a_@NPs, the formulations were negatively stained with uranyl acetate and then imaged by TEM (Figure 1d). As reported in Figure 1d, mMSC-TPCS_2a_@NPs showed a spherical shape with an outer membrane shell. The sizes of TPCS_2a_@NPs and mMSC-TPCS_2a_@NPs detected by TEM were respectively 31.3 ± 4.01 nm and 43.6 ± 7.30 nm, which were smaller than what was observed before, probably because of the solvation layer detected by DLS measurement. The ~12 nm size variation between the two formulation was consistent with the thickness of the phospholipid bilayer. The ZP slightly decreased from −10.56 ± 0.22 mV for TPCS_2a_@NPs to −16.06 ± 0.59 mV for mMSC-TPCS_2a_@NPs, thanks to the additional negative charges introduced by the MSC membranes (Figure 1b). The mild negative ZP observed for non-coated TPCS_2a_@NPs could be attributed to the presence of the carboxylic groups of the PLGA polymer used to assemble the NPs together with the negative charges of the sulfated group of TPCS_2a_. As expected, we did not observe a high negative ZP for uncoated PLGA NPs since the measurement was performed in HEPES buffer, pH 7.4 [40].

Finally, SDS-PAGE was performed to analyze the protein profile of mMSC-TPCS_2a_@NPs using both ASC52telo whole cells lysate and isolated mMSCs, as controls. As expected, the protein pattern observed in the silver-stained polyacrylamide gel (Figure 1c) for mMSC-TPCS_2a_@NPs was very similar to that of the mMSCs, suggesting the protein composition of extracted membranes was retained during the NP coating procedure. On the contrary, ASC52telo whole cells lysate showed a different protein composition.

**Figure 2:**
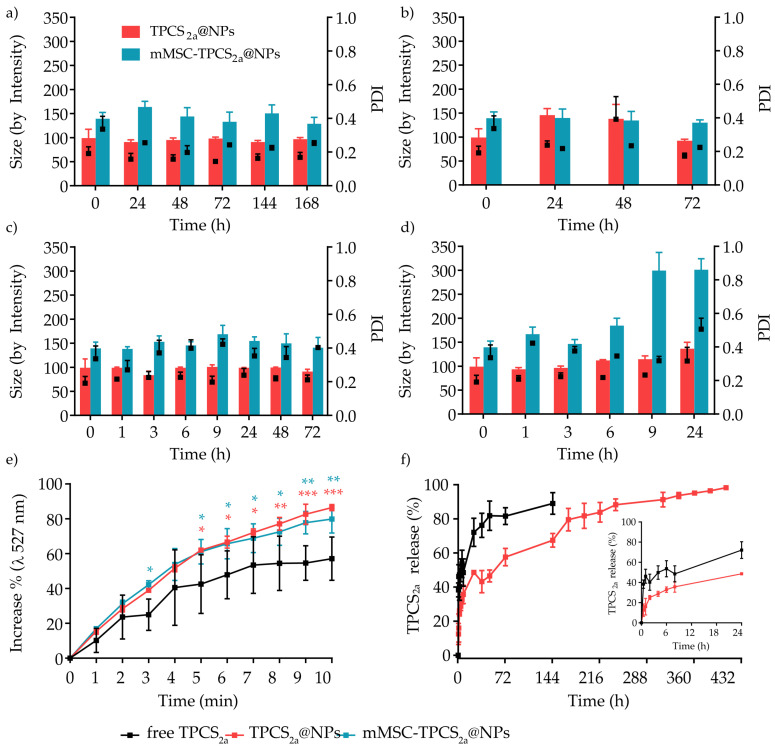
Physico-chemical characterization of TPCS_2a_@NPs and mMSC-TPCS_2a_@NPs. Stability of TPCS_2a_@NPs and mMSC-TPCS_2a_@NPs in (**a**) H_2_O at 4 °C, (**b**) H_2_O at 37 °C, (**c**) DMEM + 5% FBS at 4 °C and (**d**) DMEM + 5% FBS at 37 °C. (**e**) Monitoring of singlet oxygen (^1^O_2_) generation with an SOSG probe in the presence of free TPCS_2a_ solution or TPCS_2a_@NP and mMSC-TPCS_2a_@NP suspensions. Data are expressed as mean percentage ± SD of at least two independent experiments. Statistical analysis (two-way ANOVA, NPs vs. free TPCS_2a_): * *p* < 0.05; ** *p* < 0.01; *** *p* < 0.001. (**f**) Release profiles of free TPCS_2a_ or TPCS_2a_ from TPCS_2a_@NPs over a period of 17 days. Data are expressed as mean percentage ± SD of three independent experiments.

Another crucial parameter to consider when formulating NPs is their colloidal stability in the physiological milieu, since NP aggregation would affect their in vivo biodistribution, safety profile, and efficacy [39]. Therefore, the stability of TPCS_2a_@NPs and mMSC-TPCS_2a_@NPs was assessed by monitoring over time their hydrodynamic diameter and PDI after dilution in H_2_O and maintenance at 4 °C, or after dilution in DMEM culture medium supplemented with 5% of FBS (*v*/*v*) and incubation at 37 °C, to mimic storage and physiological-simulated conditions, respectively. The addition to the culture medium of higher percentages of serum was hampered by the protein interference with the DLS analysis, which completely hid the NP peak. After dilution in H_2_O and storage at 4 °C, TPCS_2a_@NPs and mMSC-TPCS_2a_@NPs were found to be stable up to 7 days (Figure 2a) since their size and PDI did not change considerably. In contrast, when maintained in water at 37 °C, all formulations were stable up to 72 h (Figure 2b), followed by a significant and rapid increase in size, doubling from ~139 to over 300 nm for mMSC-TPCS_2a_@NPs, indicating that aggregation phenomena started to occur for the coated NPs after this time. When diluted in DMEM + 5% FBS, the diameter of TPCS_2a_@NPs and mMSC-TPCS_2a_@NPs remained almost stable at 4 °C (Figure 2c), while it increased in a more controlled manner for TPCS_2a_@NPs as compared to mMSC-TPCS_2a_@NPs incubated at 37 °C, with PDIs remaining mostly unaltered for both NP formulations (Figure 2d).

To enhance the long-term stability and storage of the NPs and avoid hydrolysis of the polymer matrix, which can lead to drug leakage and the formation of unwanted degradation products [39], additional lyophilization studies were carried out by varying the percentage (1–10% *w*/*v* range) and type of cryoprotectant used (i.e., sucrose, glucose, trehalose, and dextrose). In the tested conditions, after the lyophilization and resuspension processes TPCS_2a_@NPs maintained a similar diameter and PDI to those of the pre-lyophilized sample (Supplementary Figure S1), regardless of the type of sugar or its concentration. Only dextran at 10% *w*/*v* concentration showed a relevant increase in size from 99 nm to almost 200 nm. On the other hand, in the absence of any cryoprotectant the size of TPCS_2a_@NPs increased from 99 to 315 nm, resulting in NPs with a very broad particle size distribution, as supported by the PDI value of about 0.8. Importantly, the EE% did not vary before and after the lyophilization process.

Once the stability of the particles was assessed, UV-Vis spectrophotometric and spectrofluorimetric analyses were performed to predict the photodynamic efficiency of our formulations. The photophysical properties of TPCS_2a_ encapsulated in the uncoated or coated NPs vs. the free form were compared at a final TPCS_2a_ concentration of 2.5 μM, using H_2_O or DMEM added with 10% of serum as solvents at a temperature of 37 °C. By looking at the spectra of free TPCS_2a_ in H_2_O over 24 h (Supplementary Figure S2(a1)), it is possible to observe a significant decrease in Soret band absorbance (around 400 nm), owing to the tendency of TPCS_2a_ to aggregate in aqueous media [41], and a resulting red shift (λ_em_ from 656 to 659 nm) in the fluorescence spectra over time (Supplementary Figure S2(a2)). On the contrary, when TPCS_2a_ was encapsulated either in TPCS_2a_@NPs or mMSC-TPCS_2a_@NPs and particles were suspended in H_2_O, the decrease in absorption and fluorescence was significantly lower than that of free TPCS_2a_ and characterized by the absence of the spectra profile modification or the red shift (Supplementary Figure S2(a3–6)). However, a general decrease in the absorbance and the fluorescence was still observed. We hypothesized the sedimentation of a small fraction of TPCS_2a_@NPs or mMSC-TPCS_2a_@NPs in water over time as the determinant of reduced absorbance/fluorescence rather than aggregation of the PS molecules inside the NPs, which would have resulted in a variation of the spectrum profiles. Complete stability for the duration of the experiment was observed in DMEM in the presence of 10% serum for the NP formulations, suggesting no modifications of the photophysical properties of the PS when encapsulated in both nanocarrier types (Supplementary Figure S2(b3–6)). Both the shape and the intensity of the absorption and fluorescence spectra were comparable to those of the free drug (Supplementary Figure S2(b1,2)). Further evidence that TPCS_2a_ was not aggregated when encapsulated in NPs included the presence of a broad shoulder and the splitting of the Soret band around 400 nm in the absorption spectra, both of which are suggestive of the monomeric state of TPCS_2a_ (Supplementary Figure S2(b3,5)) [42].

Since the production of ^1^O_2_ is crucial for PDT effectiveness, we evaluated its generation after red light irradiation (600–800 nm) of free TPCS_2a_, TPCS_2a_@NPs, and mMSC-TPCS_2a_@NPs diluted in aqueous medium and added to the singlet oxygen sensor green (SOSG) probe. The probe is essentially composed of a fluorochrome whose fluorescence is quenched by an anthracene moiety. The ^1^O_2_ generated after PS irradiation interacts with the anthracene to form an oxygen adduct, restoring the fluorescence, and the increase in fluorescence at the wavelength of 527 nm is used to measure this ^1^O_2_-specific reaction. As shown in Figure 2e, which reported the increase percentage in SOSG fluorescence, TPCS_2a_@NPs and mMSC-TPCS_2a_@NPs demonstrated a significantly higher ^1^O_2_ generation with final increases of 22% and 29%, respectively, as compared to the free drug. The lower ^1^O_2_ generated by free TPCS_2a_ as compared to the NPs can be mainly ascribed to the aggregation state of the molecules in the aqueous environment, while the encapsulation of TPCS_2a_ in NPs reduced the interaction with water molecules, thus limiting the drug aggregation and enabling a higher ^1^O_2_ production.

Then, we investigated by dialysis method the cumulative drug release from TPCS_2a_@NPs in phosphate buffer (10 mM, pH 7.4) added with 0.05% *w*/*v* of Tween 80, at 37 °C. The results displayed in Figure 2f indicated a burst release probably ascribable to the drug dissociation from the PLGA surface. Indeed, it is possible that TPCS_2a_ was not only encapsulated in PLGA NPs, but that it also formed an outer layer owing to the simple association with the NP surface by hydrophobic interaction. As shown in Supplementary Figure S3, the initial fast release (24 h) could be well described by the Weibull model (y = −0.12x + 1.817, R^2^ = 0.9899) and was followed by a controlled release of entrapped TPCS_2a_ over 21 days, caused by the concomitant degradation of the PLGA matrix, according to the Korsmeyer–Peppas model (y = 0.3437x + 1.0063, R2 = 0.9820) [43,44]. As control, free drug release from the dialysis bag was also monitored under identical conditions. The drug tendency to aggregate slowed down the diffusion through the membrane, thus resulting in almost 90% of TPCS_2a_ released over 6 days, while the remaining drug was found as a precipitate inside the dialysis membrane.

Before moving to the in vitro studies, we checked whether the mMSC coating was preserved after mMSC-TPCS_2a_@NP interaction with cells. To this aim, mMSCs were stained with the lipophilic fluorophore DiO and then used to coat TPCS_2a_@NPs. DiO-labeled mMSC-TPCS_2a_@NPs were incubated with MCF7 cells and imaged by a confocal laser-scanning microscope to assess the colocalization of the DiO-labeled mMSC green signal with the fluorescence red emission of TPCS_2a_ entrapped in the NPs. As shown in Supplementary Figure S4a, after 2 h incubation of MCF7 cells with mMSC-TPCS_2a_@NPs it was possible to observe some overlap of DiO and TPCS_2a_ signals. Of note, the colocalization was still consistent at 24 h of incubation (Supplementary Figure S4b), where the signal from the internalized particles was much higher, suggesting that mMSC-TPCS_2a_@NPs preserve their camouflage for a prolonged time.

### 3.2. In Vitro NP Capture by Human Macrophages

**Figure 3:**
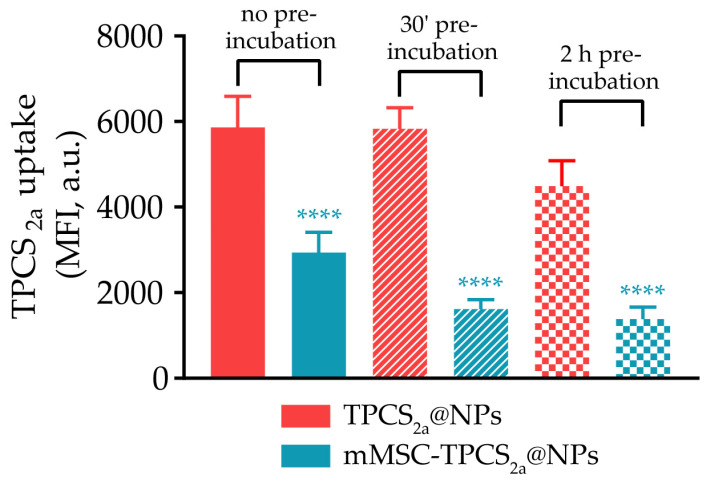
NP capture by human macrophages. Flow cytometry data showing the mean fluorescence intensity (MFI) of TPCS_2a_ in human macrophages incubated with 1 μM TPCS_2a_@NPs or mMSC-TPCS_2a_@NPs and incubated at 37 °C for 2 h. In some cases, NPs were pre-incubated with human serum (HS) for 30 min or 2 h at 37 °C and then placed in contact with cells. Data are expressed as mean percentage ± SD of at least three independent experiments carried out in triplicate. Statistical analysis (one-way ANOVA): **** *p* < 0.0001.

It is well established that systemic injection of nanocarriers can trigger different host defense responses such as opsonization and phagocytosis by the mononuclear phagocytic system (MPS) component. In fact, plasma proteins can adsorb on NPs via hydrophobic or electrostatic interactions, thus forming a protein corona that causes the recognition and elimination of the particles by phagocytic cells, seriously affecting NP/drug biodistribution [45]. The rationale behind the production of our biomimetic NPs was the evidence that coating NPs with cell membranes increases their circulation time and improves their therapeutic efficacy [46,47]. This ability seems to rely on specific carbohydrates and lipids displayed at the cell membrane outer surface, which dictates unique interactions with plasma proteins in vivo. Consequently, a different protein corona forms around the biomimetic NPs, which minimizes the recognition by immune cells [48]. To assess that the use of mMSCs as a biomimetic coating of NPs effectively affects the extent of NP phagocytosis, we compared the internalization of uncoated TPCS_2a_@NPs and coated mMSC-TPCS_2a_@NPs by human macrophages after 2 h of incubation in serum-free culture medium. Macrophages were derived from monocytes isolated from the buffy coats of healthy donors. Furthermore, to gain more insight on the impact of the protein corona on NP uptake by macrophages, particles were pre-incubated for 30 min or 2 h with human serum (HS) before the uptake studies. The NP association with macrophages was monitored by flow cytometry and expressed as the mean fluorescence intensity (MFI) of TPCS_2a_ taken up by cells. As shown in Figure 3, NP internalization by macrophages was significantly decreased in the case of mMSC-TPCS_2a_@NPs as compared to uncoated TPCS_2a_@NPs. The MFI reduction was even more pronounced when NPs were pre-incubated with HS for 30 min or 2 h, confirming that the protein corona that forms around mMSC-TPCS_2a_@NPs is capable of further reducing NP phagocytosis. Collectively, these data showed that the coating of TPCS_2a_@NPs with MSC membranes modulates the protein corona, and it is effective in reducing NP uptake by macrophages, which may prolong the circulation time after in vivo administration.

### 3.3. In Vitro Uptake and Localization Studies in Monolayered Breast Cancer Cells

**Figure 4:**
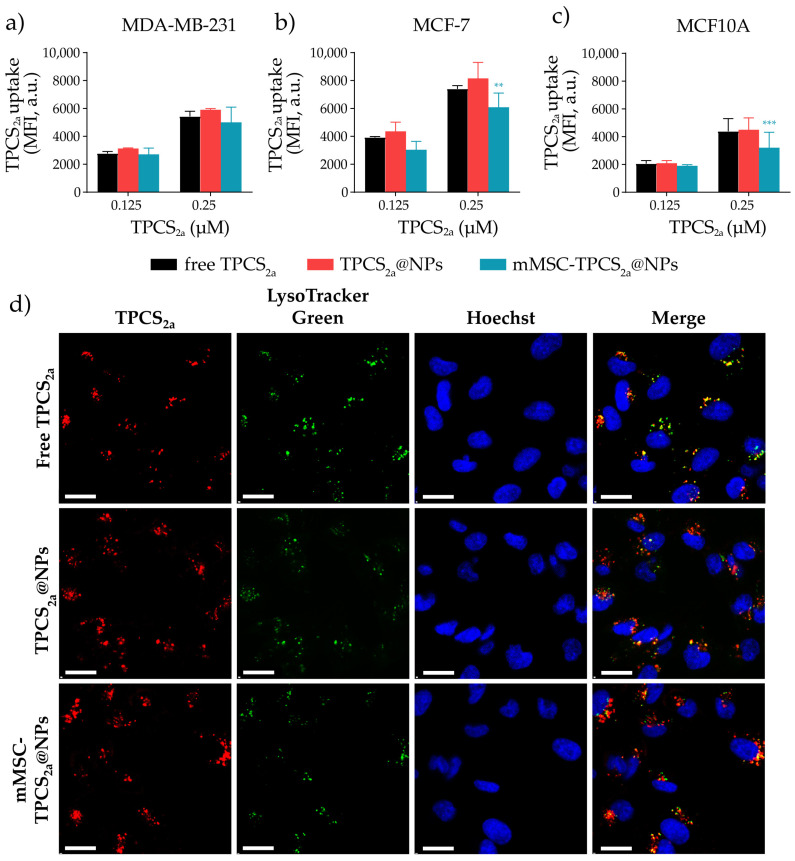
In vitro uptake and intracellular localization of TPCS_2a_. Cell uptake measured by flow cytometry after incubation of MDA-MB-231 (**a**), MCF7 (**b**), and MCF10A (**c**) cells for 24 h with free TPCS_2a_, TPCS_2a_@NPs, or mMSC-TPCS_2a_@NPs. Data are expressed as mean percentage ± SD of at least two independent experiments carried out in triplicate. Statistical analysis (two-way ANOVA, NPs versus free TPCS2a): ** *p* < 0.01; *** *p* < 0.001. (**d**) Confocal microscopy images of MDA-MB-231 cells after incubation with free TPCS_2a_, TPCS_2a_@NPs, or mMSC-TPCS_2a_@NPs showing TPCS_2a_ (red), LysoTracker Green lysosome probe (green), Hoechst 33342 nuclei staining (blue). Scale bars: 20 μm.

It is well established that the success of PDT outcome is strictly related to the extent of PS uptake by tumor cells, while the subcellular localization significantly affects the cell death mechanism [49,50]. Therefore, the in vitro uptake of freeTPCS_2a_, TPCS_2a_@NPs, or MSC-TPCS_2a_@NPs by breast cancer models such as MCF7 adenocarcinoma and MDA-MB-231 triple negative BC cells, and in the non-malignant mammary epithelial MCF10A cell line, was evaluated by flow cytometric studies. As shown in Figure 4a,b, TPCS_2a_ association with tumor cells increased in a dose-dependent manner for all TPCS_2a_ formulations and cell lines tested. TPCS_2a_ internalization was remarkably higher in MCF7 as compared to MDA-MB-231 cells at both concentrations used. Importantly, while the uptake of both TPCS_2a_@NPs and mMSC-TPCS_2a_@NPs by MDA-MB-231 cells was similar to that of the free drug (*p* > 0.05), a statistically significant lower internalization of mMSC-TPCS_2a_@NPs by MCF7 cells was observed at the higher concentration tested. Despite that, the uptake of coated NPs detected in both lines was remarkably high. Interestingly, the internalization of TPCS_2a_ by the non-tumorigenic MCF10A breast cell line was reduced when compared to the other BC cell lines in all the treatment options (Figure 4c). TPCS_2a_@NP and mMSC-TPCS_2a_@NP internalization by MCF10A cells were 24% and 35% lower as compared to MDA-MB-231 cells, and 45% and 47% lower than that observed in MCF7 cells, respectively, thus supporting the use of the biomimetic coating for reducing NP uptake by non-malignant cells.

The intracellular localization of TPCS_2a_ NPs in comparison with free TPCS_2a_ was assessed by confocal microscopy studies in MDA-MB-231 cells incubated with TPCS_2a_, TPCS_2a_@NPs, or mMSC-TPCS_2a_@NPs for 24 h. As shown in Figure 4d, we observed partial colocalization between the red fluorescence of TPCS_2a_ and the green fluorescence of the specific lysosomal probe LysoTracker Green in all analyzed samples. This agreed with what was already reported by us [37] and others [51] concerning the lysosomal accumulation of TPCS_2a_ and polymeric TPCS_2a_-loaded NPs.

### 3.4. In Vitro Cytotoxicity in Monolayered Breast Cancer Cells

**Figure 5:**
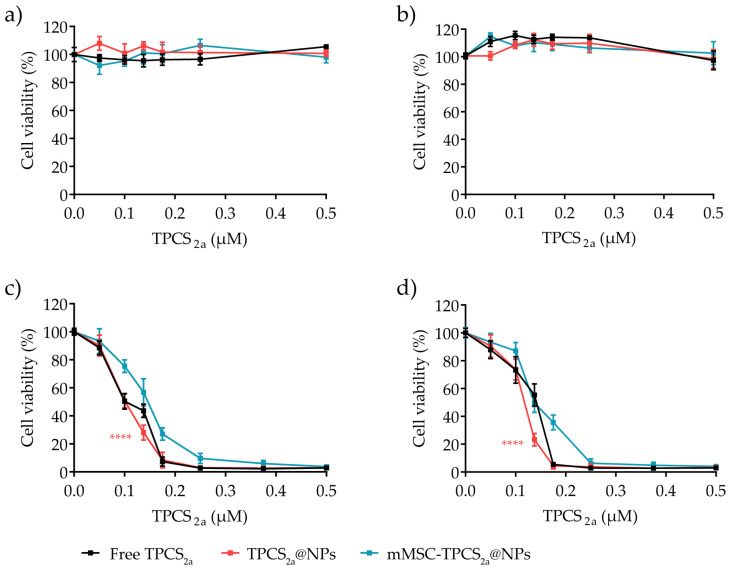
Dark toxicity and phototoxicity of TPCS_2a_ formulations toward monolayered breast cancer cells. Cytotoxicity profiles of MDA-MB-231 (**a**,**c**) and MCF7 (**b**,**d**) cells exposed to increasing concentrations of TPCS_2a_, TPCS_2a_@NPs, or mMSC-TPCS_2a_@NPs. Cytotoxicity of TPCS_2a_ was assessed by MTS assay after incubation of cells in the dark (**a**,**b**) as well as after exposure to 10 J/cm^2^ of red light (**c**,**d**), and an additional 24 h incubation in a drug-free medium. Data are expressed as mean ± SD of at least three independent experiments carried out in triplicate. Statistical analysis (two-way ANOVA, free TPCS_2a_ versus TPCS_2a_@NPs or mMSC-TPCS_2a_@NPs): **** *p* < 0.0001.

One of the key advantages of PDT over other chemotherapies resides in the lack of toxicity of PSs in the absence of light irradiation. To confirm at the same time the safety and activity of our formulations we investigated the dark and light cytotoxicity of TPCS_2a_ NPs in MDA-MB-231 and MCF7 cells at increasing concentrations of TPCS_2a_ (0.1–0.5 µM range). Cells were incubated with the TPCS_2a_ formulations for 24 h and grown for additional 24 h in drug/NP-free medium before measuring viability by the MTS assay. As shown in Figure 5a,b, no cytotoxicity was observed in MDA-MB-231 and MCF7 cells or in the presence of free TPCS_2a_ or PS-loaded NPs when the experiment was performed in the dark. In the phototoxicity studies, after 24 h incubation with NP suspensions, cells were irradiated with a 10 J/cm^2^ total dose of red light, and cell viability was measured after an additional 24 h of cell incubation in a drug-free medium. As expected, TPCS_2a_ activation with red light irradiation induced an efficient and dose-dependent decrease in the cell viability in both cell lines for all tested formulations (Figure 5c,d). Indeed, both TPCS_2a_@NPs and mMSC-TPCS_2a_@NPs reduced cell viability to an extent similar to the free drug, their profiles of the dose-response curve were comparable and all IC_50_ values < 0.15 µM (Table 1). Taken together, these results importantly highlight that the encapsulation in a drug delivery system does not affect TPCS_2a_ photodynamic efficiency, in agreement with the optimal singlet oxygen production previously measured in an aqueous environment (Figure 2e). As expected, phototoxicity data mostly correlated with the intracellular uptake of TPCS_2a_ (Figure 4a,b), with the IC_50_ values being TPCS_2a_@NPs < TPCS_2a_ < mMSC-TPCS_2a_@NPs in each cell line (Table 1). Even though the IC_50_ values observed were quite similar in all the tested conditions, we would like to stress the fact that nanotechnologies positively impact on photosensitizer (PS) and, in this specific case, TPCS_2a_ performances by increasing its solubility and limiting its aggregation tendency [52]. Thus, TPCS_2a_ encapsulation in NPs will offer the advantages of increased photochemical efficiency combined with higher localization in tumors by the EPR effect after administration [53].

Independent of the absolute amount of internalized TPCS_2a_, the IC_50_ values observed in MDA-MB-231 cells for free TPCS_2a_, TPCS_2a_@NPs, and mMSC-TPCS_2a_@NPs treatments were slightly lower with respect to that measured for MCF7 cells. Indeed, despite the higher uptake of TPCS_2a_ formulations in MCF7 cells as compared to MDA-MB-231 cells, the latter was proved to be slightly more sensitive to the oxidative stress/damages induced by the PDT treatment. These results well correlated with previous studies that reported a different susceptibility of the two cell lines to PDT treatment [54]. Kwiatkowska and coworkers reported that, as opposed to MDA-MB-231 cells, the MCF7 cell line expressed the antioxidant protein glutathione peroxidase 4 (GPX-4), an enzyme capable of protecting cells from lipid peroxidation damages. The lack of GPX-4 seems to reduce the capacity of the cells to prevent damages induced by ^1^O_2_ and other ROS released during PDT, leading to increased cell death [55]. Importantly, the elevated efficacy of PDT treatment with TPCS_2a_ nanoformulations on MDA-MB-231 cells using PS concentrations in the nanomolar range confirmed once again the potential of PDT treatment for TNBC subtypes that are highly resistant and minimally responsive to conventional therapies [34,37,38].

### 3.5. In Vitro Uptake and Cytotoxicity in 3D Spheroids

**Figure 6:**
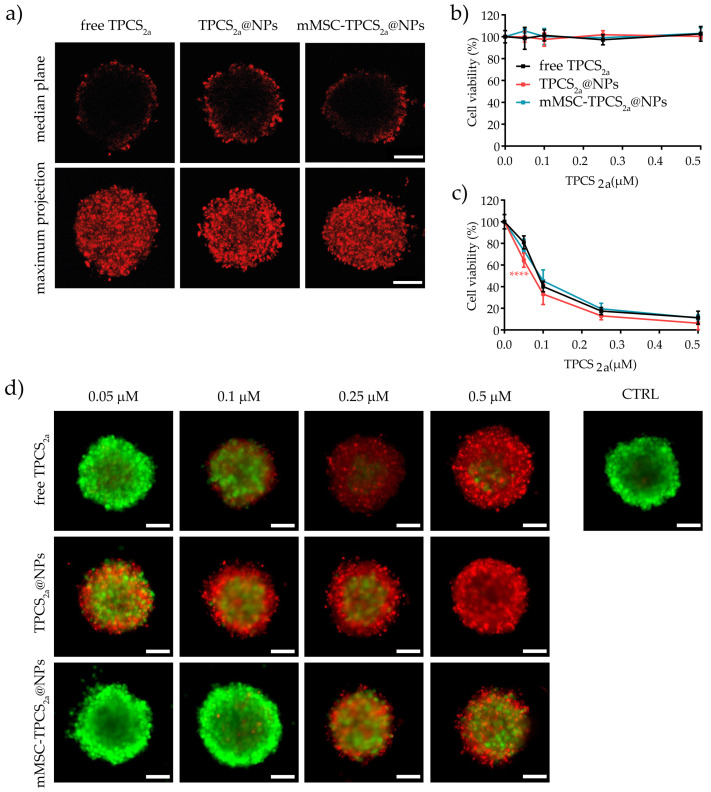
In vitro uptake and cytotoxicity of TPCS_2a_ formulations in MDA-MB-231 spheroids. (**a**) Confocal microscopy images of spheroids treated for 24 h with free TPCS_2a_, TPCS_2a_@NPs, and mMSC-TPCS_2a_@NPs at 1 μM TPCS_2a_ equivalent concentration. The images represent TPCS_2a_ fluorescence distribution at the equatorial plane of each spheroid and the maximum projection of 20 different focal planes superimposed. Scale bar: 100 μm. (**b**) Cytotoxicity in spheroids was assessed after 24 h of incubation in the dark or (**c**) exposure to 10 J/cm^2^ of red light, and an additional 24 h incubation in drug-free medium. Cell viability was measured by CellTiter-Glo 3D Assay. Data are expressed as mean ± SD of at least two independent experiments carried out in quadruplicate. Statistical analysis (two-way ANOVA, free TPCS_2a_ versus TPCS_2a_@NPs and mMSC-TPCS_2a_@NPs): **** *p* < 0.0001. (**d**) Selected images of spheroids stained with the LIVE/DEAD kit and acquired 24 h after irradiation with 10 J/cm^2^ of red light. Scale bar: 100 μm.

The above-described findings collectively demonstrated that the biomimetic NPs we generated represent an efficient TPCS_2a_ delivery system directed toward tumor cells, endowed with elevated photodynamic activity and able to counteract macrophage capture at least in in vitro cell monolayers. However, the use of three-dimensional in vitro cancer spheroids can better mimic the in vivo tumor microenvironment and cell–cell interaction, providing a more sophisticated model for evaluating the diffusion of the PSs and the efficacy of PDT [56]. Therefore, we selected TNBC MDA-MB-231 cells to produce spheroids and gain more insights on the penetration and diffusion capacity of our biomimetic NPs. Four days old spheroids were incubated with free TPCS_2a_, TPCS_2a_@NPs, and mMSC-TPCS_2a_@NPs at 1 μM TPCS_2a_ equivalent concentration, and the uptake of the chlorin was monitored by confocal laser-scanning microscopy (Figure 6a). The TPCS_2a_ fluorescence signal acquired at the median plane of the spheroid showed penetration of TPCS_2a_ exclusively in the outer cell layers for all the three formulations, in agreement with what was reported by us [57,58] and others [59] on PS uptake in a 3D tumor model. Importantly, the use of coated or uncoated NPs to deliver TPCS_2a_ significantly increased the penetration in the 3D structure, as is clearly visible in Figure 6a. The maximum projection images obtained from the superimposition of at least 20 different focal plans showed a homogeneous distribution of TPCS_2a_ in the outer cell layer and again superior accumulation of TPCS_2a_@NPs and mMSC-TPCS_2a_@NPs in the spheroids with respect to free TPCS_2a_. Of note, the use of spheroids as a solid tumor model allowed us to highlight the improved penetration and accumulation of TPCS_2a_ formulated in an NP as compared to the free drug, which could not be appreciated in monolayered MDA-MB-231 cells (Figure 4a).

Cell viability experiments on MDA-MB-231 spheroids incubated with increasing concentrations of free TPCS_2a_, TPCS_2a_@NPs, and mMSC-TPCS_2a_@NPs in the absence of light irradiation (Figure 6b) confirmed the lack of dark toxicity observed in cell monolayers (Figure 5a). For phototoxicity experiments, spheroids incubated for 24 h with the TPCS_2a_ formulations were exposed to 10 J/cm^2^ of red light, and cell viability was measured with the 3DGlo assay after an additional 24 h of spheroid The funding data are reported correctly s release in drug-free medium. As shown in Figure 6c, irradiation caused a dose-dependent decrease in cell viability with dose-response curves quite superimposable among the three TPCS_2a_ formulations, despite the reduced uptake observed qualitatively for the PS delivered as free drug or encapsulated within a nanoparticle.

To estimate the extent of cell death induced upon light irradiation in the different topological regions of TPCS_2a_-treated spheroids, in correlation with PS localization, the LIVE/DEAD assay was used to simultaneously stain live cells (calcein, green signal) and dead cells (ethidium homodimer-1, red signal) (Figure 6d). As expected, the number of dead cells increased in a dose-dependent manner, although the extent of death among the three different TPCS_2a_ formulations was substantially different. Indeed, contrary to the data obtained with the 3DGlo assay, which measured cell viability reduction on the base of ATP released in the culture medium, where no differences in the cytotoxicity were observed among the formulations, LIVE/DEAD staining revealed a variable number of dead cells according to TPCS_2a_@NPs > free TPCS_2a_ > mMSC-TPCS_2a_@NPs. Importantly, it should be noted that although at low TPCS_2a_ concentrations (0.05–0.1 µM) the cell death was effectively restricted to the periphery of the spheroids, well correlating with the uptake results (Figure 6a), at higher TPCS_2a_ concentrations dead cells could be clearly visualized in the inner part of the spheroids. The induction of cell death in a spheroid region devoid of PS uptake could be ascribed to a bystander effect. For example, Dahle and coworkers reported on a possible combination of direct cellular damages and cellular death propagation after PDT treatment [57,60]. Since a number of molecular players and pathways have been identified as mediators of the bystander effects, further investigations are needed to better assess which cellular mechanisms are involved.

## 4. Conclusions

We herein reported the synthesis and in vitro characterization of a novel tumor-targeting biomimetic nanoplatform for the PDT treatment of breast cancer. TPCS_2a_-loaded PLGA NPs (TPCS_2a_@NPs) with a size and PDI of 98.89 ± 18.56 nm and 0.191 ± 0.04, respectively, were successfully prepared by a microfluidic technique, showing high reproducibility, an encapsulation efficiency of 82%, and a sustained drug release over 18 days. TPCS_2a_@NPs were coated with MSC-derived membranes through an efficient and easy procedure yielding a colloidal stable system (mMSC-TPCS_2a_@NPs) with a particle diameter of 139.31 ± 12.94 nm. The NP formulations revealed a higher production of singlet oxygen, with 22% and 29% higher levels for TPCS_2a_@NPs and mMSC-TPCS_2a_@NPs at the final time point as compared to the free drug, thus confirming the polymeric NPs as an optimal delivery vehicle for PSs. Moreover, the membrane-coated NPs displayed a macrophage uptake reduced by more than 50% with respect to their uncoated counterpart. The in vitro uptake studies revealed that mMSC-TPCS_2a_@NPs were internalized to a higher extent by BC than by non-tumoral breast cells and showed IC_50_ after light irradiation in the nanomolar range (<145 nM) in both MDA-MB-231 and MCF7 BC cell lines. Moreover, no cytotoxicity was detected when cells were exposed to the NP formulation in the dark conditions, supporting the safety profile of this therapeutic approach in healthy tissues. Finally, TPCS_2a_@NPs and mMSC-TPCS_2a_@NPs showed good diffusion in MDA-MB-231 3D spheroids, which was higher than the free drug. The DEAD/LIVE assay further confirmed the ability of both formulations to cause cell death in the inner part of the spheroids, probably through both direct and bystander effects. All things considered, our work suggests the potential of mMSC-coated PLGA NPs as an efficient biocompatible vehicle for photosensitizers. These results point out PDT therapy as promising treatment, especially for highly resistant TNBC.

## Figures and Tables

**Table 1:** IC_50_ values calculated from the phototoxicity curves of MDA-MB-231 and MCF7 cells exposed to increasing concentrations of equimolar TPCS_2a_ formulations.

Treatment	MDA-MB-231 IC_50_ (μM)	MCF-7 IC_50_ (μM)
Free TPCS_2a_	0.1055	0.1315
TPCS_2a_@NPs	0.1009	0.1160
mMSC-TPCS_2a_@NPs	0.1392	0.1446

## Data Availability

The data presented in this study are available on request from the corresponding authors.

## Supplementary Materials

The following supporting information can be downloaded at: https://www.mdpi.com/article/10.3390/pharmaceutics15061654/s1, Figure S1: Lyophilization studies; Figure S2: Absorption and fluorescence spectra of TPCS2a formulations in different solvents; Figure S3: Drug release plots for TPCS2a@NPs; Figure S4: Endocytosis of mMSC-TPCS2a@NPs.
