# Supplementary material for: Mesenchymal Stem Cell Membrane-Coated TPCS2a-Loaded Nanoparticles for Breast Cancer Photodynamic Therapy
Source: Pharmaceutics. 2023 Jun 4;15(6):1654. doi: 10.3390/pharmaceutics15061654 (PMC10302938; doi:10.3390/pharmaceutics15061654)
Supplement: Supplementary file 1 [file pharmaceutics-15-01654-s001.zip › pharmaceutics-2405367-supplementary.pdf]

Supporting Information

Mesenchymal Stem Cell Membrane-Coated $TPCS_{2a}$-Loaded Nanoparticles for Breast Cancer Photodynamic Therapy

Greta Avancini [1,†], Luca Menilli [1,†], Adele Visentin [2], Celeste Milani [1], Francesca Mastrotto [2,*,‡] and Francesca Moret [1,*,‡]

[1] Department of Biology, University of Padova, Via Ugo Bassi 58/B, 35131 Padova, Italy; greta.avancini@phd.unipd.it (G.A.); luca.menilli@unipd.it (L.M.); celeste.milani@phd.unipd.it (C.M.)

[2] Department of Pharmaceutical and Pharmacological Sciences, University of Padova, Via F. Marzolo 5, 35131 Padova, Italy; adelevisentin96@gmail.com

* Correspondence: francesca.mastrotto@unipd.it (F.M.); francesca.moret@unipd.it (F.M.)

† These authors equally contributed to this paper.

‡ These authors equally contributed as senior and corresponding authors.

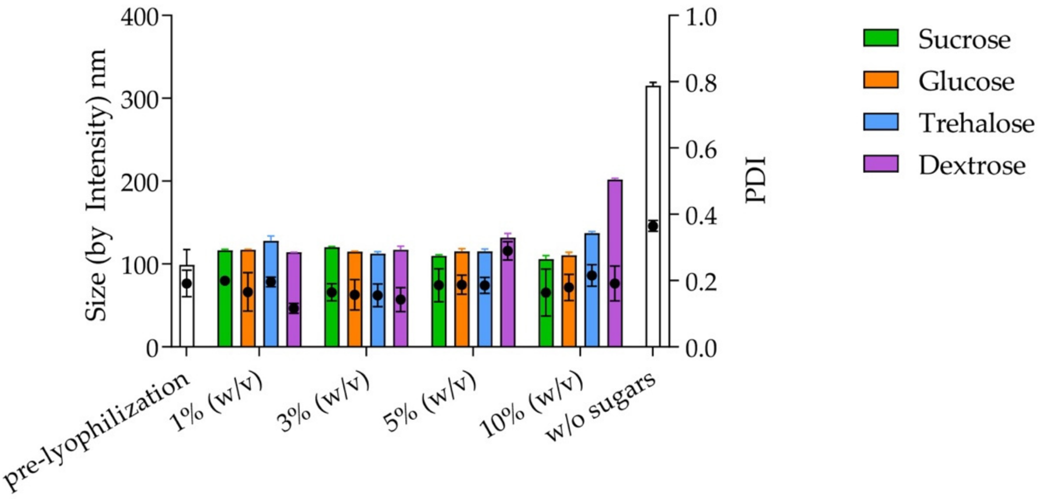

Figure S1. Lyophilization studies. Size (expressed by Intensity) and PDI of $TPCS_{2a}$@NPs re-suspended in water after freeze-drying with different percentages (w/v) of sucrose, glucose, trehalose and dextrose as cryoprotectants measured by dynamic light scattering (DLS).

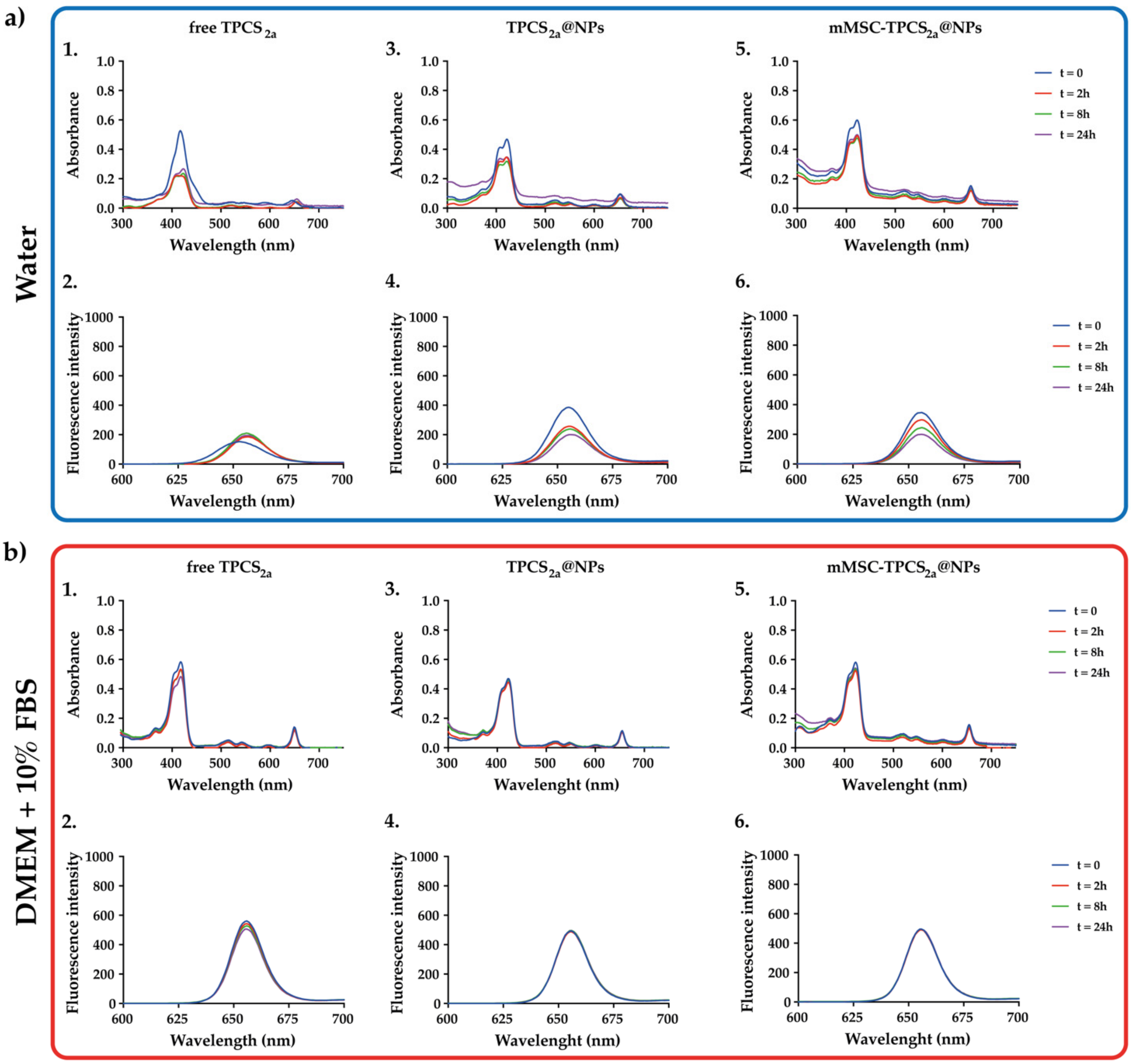

Figure S2. Absorption and fluorescence spectra of TPCS$_{2a}$ formulations in different solvents. Absorption and fluorescence spectra of TPCS$_{2a}$ in the free form (1,2), TPCS$_{2a}$@NPs (3,4) and mMSC-TPCS$_{2a}$@NPs (5,6) in H_2O (a) and DMEM + 10 % of FBS (b). Spectra were acquired at time 0 (■) and after 2 (■), 8 (■) and 24 h (■) of incubation at 37 °C.

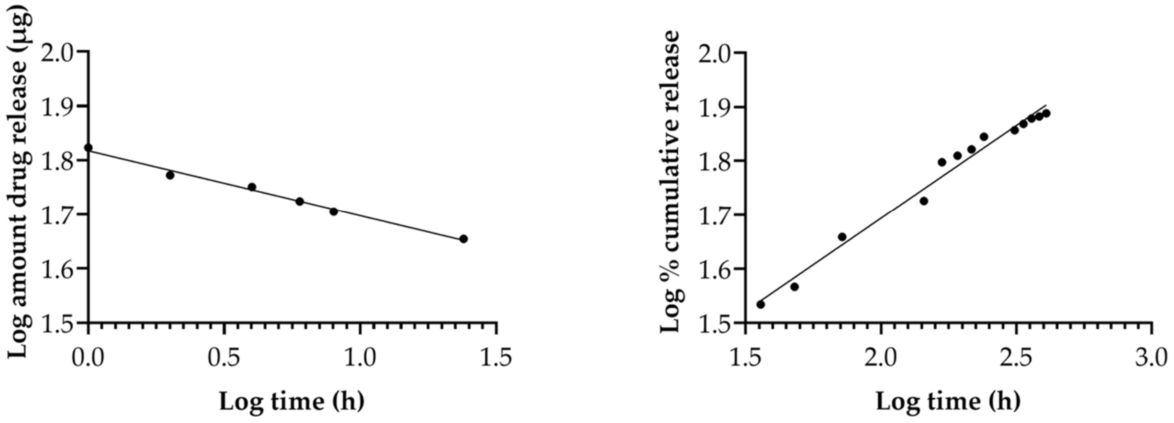

Figure S3. Drug release kinetic plot from $TPCS_{2a}$@NPs. Weibull plot for the first 24 h is displayed on the left panel and Korsmeyer-Peppas plot for the following 16 days is displayed on the right panel.

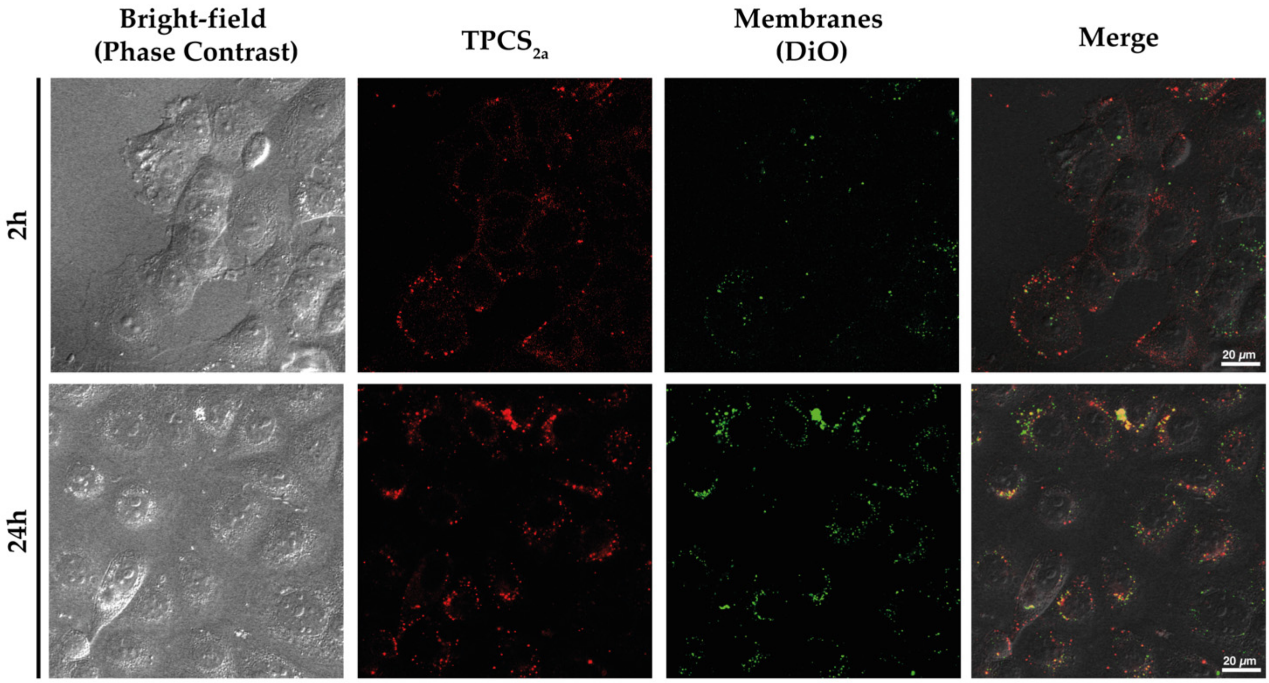

Figure S4. Endocytosis of mMSC-$TPCS_{2a}$@NPs. Confocal microscopy images of MCF7 cells incubated for 2 or 24 h with 1 μM of $TPCS_{2a}$@NPs or mMSC-$TPCS_{2a}$@NPs showing co-localization of $TPCS_{2a}$ (red) and DiO-labeled MSC membranes (green) covering the NPs. Scale bars: 20 μm.
